# Clinical presentation and long‐term outcomes of infantile hypertrophic cardiomyopathy: a European multicentre study

**DOI:** 10.1002/ehf2.13573

**Published:** 2021-09-06

**Authors:** Gabrielle Norrish, Gali Kolt, Elena Cervi, Ella Field, Kathleen Dady, Lidia Ziółkowska, Iacopo Olivotto, Silvia Favilli, Silvia Passantino, Giuseppe Limongelli, Martina Caiazza, Marta Rubino, Anwar Baban, Fabrizio Drago, Karen Mcleod, Maria Ilina, Ruth McGowan, Graham Stuart, Vinay Bhole, Orhan Uzun, Amos Wong, Laz Lazarou, Elspeth Brown, Piers E.F. Daubeney, Amrit Lota, Grazia Delle Donne, Katie Linter, Sujeev Mathur, Tara Bharucha, Satish Adwani, Jon Searle, Anca Popoiu, Caroline B. Jones, Zdenka Reinhardt, Juan Pablo Kaski

**Affiliations:** ^1^ Centre for Inherited Cardiovascular Diseases Great Ormond Street Hospital London WC1N 3JH UK; ^2^ Institute of Cardiovascular Sciences University College London London UK; ^3^ Department of Cardiology The Children's Memorial Health Institute Warsaw Poland; ^4^ Careggi University Hospital Florence Italy; ^5^ Meyer Children's Hospital Florence Italy; ^6^ Monaldi Hospital Naples Italy; ^7^ Bambino Gesu Hospital Rome Italy; ^8^ Royal Hospital for Children Glasgow UK; ^9^ West of Scotland Centre for Genomic Medicine Queen Elizabeth University Hospital Glasgow UK; ^10^ University Hospitals Bristol NHS Foundation Trust Bristol UK; ^11^ Birmingham Women and Children's NHS Foundation Trust Birmingham UK; ^12^ University Hospital of Wales Cardiff UK; ^13^ Leeds Teaching Hospitals NHS Trust Leeds UK; ^14^ Royal Brompton and Harefield Hospital, National Heart and Lung Institute Imperial College London London UK; ^15^ University Hospitals of Leicester Leicester UK; ^16^ Evelina London Children's Hospital, Guy's and St Thomas' NHS Foundation Trust London UK; ^17^ University Hospital Southampton NHS Foundation Trust Southampton UK; ^18^ Oxford University Hospitals NHS Foundation Trust Oxford UK; ^19^ Department of Pediatrics Children's Hospital ‘Louis Turcanu’, University of Medicine and Pharmacy “Victor Babes” Timisoara Timisoara Romania; ^20^ Alder Hey Children's Hospital Liverpool UK; ^21^ The Freeman Hospital Newcastle UK

**Keywords:** Infant‐onset, Hypertrophic, Cardiomyopathy, Prognosis

## Abstract

**Aims:**

Children presenting with hypertrophic cardiomyopathy (HCM) in infancy are reported to have a poor prognosis, but this heterogeneous group has not been systematically characterized. This study aimed to describe the aetiology, phenotype, and outcomes of infantile HCM in a well‐characterized multicentre European cohort.

**Methods and results:**

Of 301 children diagnosed with infantile HCM between 1987 and 2019 presenting to 17 European centres [male *n* = 187 (62.1%)], underlying aetiology was non‐syndromic (*n* = 138, 45.6%), RASopathy (*n* = 101, 33.6%), or inborn error of metabolism (IEM) (*n* = 49, 16.3%). The most common reasons for presentation were symptoms (*n* = 77, 29.3%), which were more prevalent in those with syndromic disease (*n* = 62, 61.4%, *P* < 0.001), and an isolated murmur (*n* = 75, 28.5%). One hundred and sixty‐one (53.5%) had one or more co‐morbidities. Genetic testing was performed in 163 (54.2%) patients, with a disease‐causing variant identified in 115 (70.6%). Over median follow‐up of 4.1 years, 50 (16.6%) underwent one or more surgical interventions; 15 (5.0%) had an arrhythmic event (6 in the first year of life); and 48 (15.9%) died, with an overall 5 year survival of 85%. Predictors of all‐cause mortality were an underlying diagnosis of IEM [hazard ratio (HR) 4.4, *P* = 0.070], cardiac symptoms (HR 3.2, *P* = 0.005), and impaired left ventricular systolic function (HR 3.0, *P* = 0.028).

**Conclusions:**

This large, multicentre study of infantile HCM describes a complex cohort of patients with a diverse phenotypic spectrum and clinical course. Although overall outcomes were poor, this was largely related to underlying aetiology emphasizing the importance of comprehensive aetiological investigations, including genetic testing, in infantile HCM.

## Introduction

Hypertrophic cardiomyopathy (HCM) presenting in childhood has an estimated annual incidence of 0.24–0.47 per 100 000.^1^, ^2^, ^3^ Nearly one quarter of paediatric HCM cases present in the first year of life,^4^ where the reported annual incidence is 0.51–3.2 per 100 000.^1^, ^2^, ^3^ The underlying aetiology in infant‐onset disease is more heterogeneous than that seen in later childhood, with a higher proportion of syndromic (e.g. RASopathy syndrome) or metabolic disease reported.^4^, ^5^, ^6^ Historically, sarcomeric disease has been considered to be very rare infancy due to variable and age‐related incomplete penetrance. However, more recent publications have challenged this assumption and suggested that sarcomeric disease can present in the very young.^7^, ^8^ Patients with infant‐onset HCM are recognized to have a particularly poor long‐term prognosis,^4^, ^6^, ^9^, ^10^, ^11^, ^12^, ^13^, ^14^ with mortality largely attributed to heart failure.^4^, ^6^, ^9^ However, no study to date has systematically described the aetiology, clinical features, and long‐term outcomes of infantile HCM. Understanding the spectrum and progression of disease along with an improved ability to predict long‐term outcomes would allow a more individualized approach to patient care, with important implications for clinical management. The aim of this study was to describe the aetiology, disease phenotype, and outcomes of infantile HCM in a well‐characterized multicentre European cohort.

## Methods

### Patient cohort

A retrospective, multicentre European cohort of children diagnosed with HCM in infancy between 1987 and 2019 was formed. Data were collected in 301 children from 17 European paediatric cardiac centres and included 159 patients included in previous reports.^4^, ^15^ The diagnosis of HCM was made if maximal left ventricular wall thickness (MLVWT) in any single segment was greater than two standard deviations above the body surface area‐corrected mean (*Z* score ≥ 2) and not explained by abnormal loading conditions.^16^ The diagnosis of an underlying RASopathy syndrome, inborn error of metabolism (IEM), or neuromuscular disease (HCM phenocopies) was made by the local principal investigator following systematic assessment of phenotype, biochemical, and genetic testing. In the absence of an underlying diagnosis, the aetiology was classified as non‐syndromic, in line with previous publications.^4^ Patients with confirmed disease‐causing sarcomeric variants were classified as non‐syndromic, but a separate analysis of their baseline characteristics and long‐term outcomes was performed. Eligible patients were identified by the principal investigator at each collaborating site.

### Data collection

Anonymized, non‐invasive clinical data were collected from baseline evaluation, during follow‐up, and at last clinical review, including demographics; aetiology; co‐morbidities (cardiac or extra‐cardiac); symptoms; medication; family history; genetic testing; resting and ambulatory 12 lead electrocardiograms (ECG); 2D echocardiography; and surgical or catheter interventions. Heart failure symptoms were assessed using the Ross criteria (for symptom assessment below 5 years of age)^17^ or the New York Heart Association (NYHA) functional classification (for those over 5 years of age).^17^ Echocardiographic measurements were made according to current guidelines.^16^, ^18^ Left ventricular outflow tract (LVOT) obstruction was defined as a peak instantaneous gradient ≥ 30 mmHg.^16^ Right ventricular outflow tract (RVOT) obstruction was defined as a peak instantaneous gradient ≥ 36 mmHg.^18^ Impaired left ventricular (LV) systolic function was defined as a fractional shortening (FS) ≤ 28% or ejection fraction ≤ 55%.^18^ Data were collected and verified by the principal investigator at each collaborating site.

### Outcomes

The primary patient outcomes, taken from the last clinical encounter, were all‐cause mortality [congestive cardiac failure (CCF), sudden cardiac death (SCD), other cardiovascular (CV) death, and non‐CV death] or cardiac transplantation. Secondary outcomes included major arrhythmic cardiac events (MACE), defined as SCD or an equivalent event [appropriate implantable cardioverter defibrillator (ICD) therapy, aborted cardiac arrest, or sustained ventricular tachycardia (VT) with haemodynamic compromise]^16^; atrial arrhythmias; and surgical/catheter‐based interventions. Outcomes were determined by the treating cardiologist at each site. Patients were classified as lost to follow‐up if last clinical review was more than 3 years from the end of study period (December 2019).

### Genetics

Genetic testing was performed at the treating clinician's discretion. Data were collected from patients in whom genetic testing had been performed, including date of testing; size of gene panel; and variants identified (gene and protein change). Genetic testing use across different eras was investigated: pre‐2000; 2000–2004; 2005–2009; 2010–2014; and 2015 onwards. The pathogenicity of reported variants was reclassified by the authors according to the American College of Medical Genetics and Genomics (ACMG) classification.^19^

### Statistics

Body surface area was calculated from weight.^20^ MLVWT and left atrial (LA) diameter measurements are expressed in millimetres and as body surface area‐corrected *z* scores.^21^, ^22^ Continuous variables are described as mean (± standard deviation) or median [interquartile range (IQR)], with three group comparisons conducted using analysis of variance (ANOVA) or Kruskal–Wallis tests, respectively. The distribution of categorical variables was compared using the *χ* ^2^ test or Fisher's exact test. A significance level of 0.05 was used for all comparisons.

Estimates of survival were obtained using the Kaplan–Meier product limit method. The association of clinical variables with the outcome of interest was assessed in a univariate Cox proportional hazard model. A *P* value of <0.1 was used to select variables for inclusion in a multivariable Cox proportional hazards regression model. Backwards selection techniques were used to identify variables that remained significant at 0.05 level. All statistical analyses were performed with STATA (Stata statistical software release 14; StataCorp LP, College Station, TX).

### Ethics

This study complies with the Declaration of Helsinki. Local ethical approval was obtained at each participating site with waver of informed consent for retrospective, anonymized data. The data underlying this article cannot be shared publically as consent for dissemination of patient data was not obtained.

## Results

### Demographics and presentation

Three hundred and one patients with infant‐onset HCM were identified, of whom 187 (62.1%) were male. One hundred and thirty‐eight (45.8%) had non‐syndromic HCM, 101 (33.6%) had a RASopathy, and 49 (16.3%) had an IEM. Data on aetiology and reason for presentation are shown in *Table* *1* and Supporting Information, *Table* *S1*. One hundred and sixty‐one patients (53.5%) had one or more co‐morbidities, which were more common in patients with a RASopathy (*n* = 76, 75.3%) or IEM (*n* = 35, 71.4%) (Supporting Information, *Figure* *S1*). The most common co‐morbidities varied by underlying diagnosis (Supporting Information, *Table* *S2*). Seventy‐seven (27.9%) had an additional cardiac lesion, most commonly an atrial or ventricular septal defect (*n* = 35) or valvar pulmonary stenosis (*n* = 22). Additional cardiac lesions were more common in those with a RASopathy syndrome (*n* = 42/101, 41.6%).

**Table 1:** Clinical characteristic and long‐term outcome by aetiology

		Whole cohort (*n* = 306)	Non‐syndromic (138)		RASopathy (101)	IEM (49)	*P* value	
			Non‐syndromic (138)	Non‐syndromic with genetic testing^a^ (67)			Comparison by aetiology (*n* = 288)	Comparison by aetiology using reduced cohort (*n* = 217)^a^
Male gender		187 (62.1)	88 (63.8)	43 (65.4)	66 (65.4)	28 (57.1)	0.609	0.607
FHx HCM (*n* = 294)		69 (23.5)	48 (37.5)	33 (49.3)	8 (8.1)	12 (24.5)	<0.001	<0.001
FHx SCD		23 (7.6)	14 (10.1)	7 (10.9)	2 (2)	6 (12.2)	0.026	0.048
Reason for referral (*n* = 263)	Symptomatic	77 (29.3)	18 (17.1)	12 (19.4)	35 (40.7)	21 (43.8)	<0.001	<0.001
	Antenatal diagnoses	9 (3.4)	1 (1)	1 (1.6)	5 (5.8)	2 (6.3)		
	Murmur	75 (28.5)	45 (42.9)	26 (41.9)	23 (26.7)	4 (8.3)		
	Family screening	24 (8.0)	21 (20)	14 (22.6)	0	2 (4.2)		
	Screening for associated condition	17 (6.5)	0	0	3 (3.4)	11 (22.9)		
	Other	50 (19.0)	20 (19.1)	9 (14.5)	20 (23.3)	7 (14.6)		
Co‐morbidities	Any	161 (53.5)	50 (36.2)	22 (32.8)	76 (75.3)	35 (71.4)	<0.001	<0.001
	Cardiac (*n* = 276)	77 (27.9)	30 (21.7)	13 (19.4)	42 (41.6)	4 (8.2)	<0.001	<0.001
**Initial clinical assessment**								
Ross class > 1 (*n* = 271)		99 (36.5)	24 (21.4)	11 (17.2)	47 (49.5)	24 (49.0)	<0.001	<0.001
Any cardiac symptoms		129 (42.9%)	47 (34.1)		62 (61.4)	31 (63.3)	<0.001	<0.001
Pattern of hypertrophy (*n* = 267)	ASH	118 (44.2)	80 (65.6)	53 (82.8)	26 (28.6)	6 (12.2)	<0.001	<0.001
	Concentric	64 (23.9)	16 (13.1)	4 (6.3)	22 (24.2)	24 (45.0)		
	Biventricular	85 (31.8)	22 (18.0)	7 (10.9)	40 (44.0)	18 (36.7)		
MWT (mm)	Median, IQR	9 (7,12)	9.3 (7.5,13.0)	10.5 (8, 13)	9 (7,11.8)	9 (7,11)	0.403	0.024
MWT *z* score	Mean (±)	10.8 (5.8)	10.3 (4.0)	11.6 (±5.3)	9.3 (3.1)	9.9 (3.9)	0.823	0.946
Impaired systolic function (*n* = 160)		12 (7.5)	3 (2.2)	1 (1)	1 (1)	7 (14.3)	0.001	0.001
LVOT obstruction (*n* = 265)		160 (60.6)	42 (43.8)	22 (36.1)	49 (62.8)	6 (17.1)	<0.001	<0.001
RVOT obstruction (>16 mmHg) (*n* = 155)		66 (42.6)	12 (20.3)	6 (26.1)	48 (72.7)	5 (20)	<0.001	<0.001
**Outcome**								
Died		48 (15.9)	11 (8.0)	3 (4.5)	16 (15.8)	20 (40.8)	<0.001	<0.001
	SCD	8 (2.7)	3	2	2	3		
	CCF	14 (4.7)	6	1	4	4		
	Other CV	4 (1.3)	0	0	3	0		
	Non‐CV	20 (6.6)	2	0	6	12		
	Unknown	2 (0.7)	0	0	1	1		
Transplant		6	3	1	1	2		
Mortality or transplant incidence rate/100 patient years		3.1 (95% CI 2.41–4.11)	1.33 (95% CI 0.73–2.39)	0.87 (95% CI 0.33–2.32)	2.39 (95% CI 1.47–3.91)	13.22 (95% CI 8.62–20.29)	<0.001	<0.001
Survival	1 year	86.2 (95% CI 81.7–89.7%)	94.7 (95C% CI 89.2–97.4)	96.0 (95% CI 88.3–99.2)	88.0 (95% CI 79.8–93.0)	65.1 (95%CI 50.0–76.7)		
	5 years	83.1 (95% CI 78.2% ‐ 87.0)	93.0 (95% CI 86.9–96.3)	96.0 (95% CI 88.3–99.2)	85.7 (95%CI 77.0–91.3)	57.9 (95% CI 42.5–70.6)		
	10 years	80.0 (95% CI 73.8–84.9)	90.0 (95% CI 79.8–95.2)	96.0 (95% CI 88.3–99.2)	81.1 (95%CI 69.7–88.6)	57.9 (95% CI 42.5–70.6)		

ASH, asymmetric septal hypertrophy; CCF, congestive cardiac failure; CI, confidence interval; CV, cardiovascular; FHx, family history; HCM, hypertrophic cardiomyopathy; IEM, inborn error of metabolism; IQR, interquartile range; LVOT, left ventricular outflow tract; MACE, major arrhythmic cardiac event; MWT, maximal wall thickness; NYHA, New York Heart Association; RVOT, right ventricular outflow tract; SCD, sudden cardiac death.

^a^ Patients in whom genetic testing has been performed.

### Initial clinical phenotype

Data on initial clinical assessment are shown in *Table* *1*. Cardiac medication was started in 146 (54.7%): beta‐blockers (*n* = 137, 51.3%); diuretics (*n* = 39, 14.6%); heart failure therapy including angiotensin‐converting enzyme inhibitors (ACE‐I) and diuretics (*n* = 9, 3.4%); anti‐arrhythmics (*n* = 3, 1.1%); disopyramide (*n* = 2, 0.7%); and calcium channel blockers (*n* = 1, 0.4%). Five patients required cardioactive inotropic support at presentation, of which four had an IEM.

### Genetics

**Figure 1:**
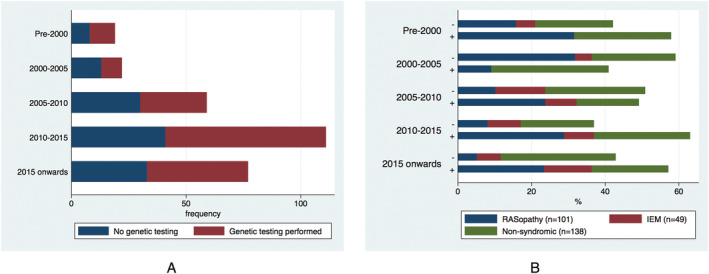
Use of genetic testing in infantile hypertrophic cardiomyopathy. (A) By era (*P* value 0.244). (B) By underlying aetiology and result. (+) represents identification of a centre‐reported variant of unknown significance or disease‐causing variant. (−) represents a negative genetic test. IEM, inborn error of metabolism.

Genetic testing strategy by era and aetiology is summarized in *Figure* *1* and Supporting Information, *Table* *S3*. Genetic testing was performed in 163 (54.2%) patients, with a disease‐causing variant identified in 115 (70.6%) (Supporting Information, *Table* *S4*). Patients with a RASopathy were more likely to undergo genetic testing (RASopathy *n* = 72, 71.3% vs. IEM *n* = 24, 49.0% vs. non‐syndromic *n* = 67, 48.6%, *P* value 0.001), but there was no difference in the overall prevalence of genetic testing over time (*Figure* *1*, Supporting Information, *Table* *S3*, *P* value 0.244).

Of patients classified as non‐syndromic, those who underwent genetic testing were more likely to have a family history of HCM (*n* = 33, 49.3% vs. *n* = 15, 24.6%, *P* value 0.004) and an asymmetric pattern of hypertrophy (*n* = 53, 82.8% vs. *n* = 27, 46.6%, *P* value < 0.001), but did not differ by the prevalence of co‐morbidities (*n* = 22, 32.8% vs. *n* = 28, 39.4%, *P* value 0.420). Of variants previously classified as pathogenic (P)/likely pathogenic (LP), after ACMG reclassification (*n* = 30), 20 variants remained pathogenic/likely pathogenic (*MYBPC3 n* = 7, *MYH7 n* = 9, *KRAS n* = 1, *LZRT1 n* = 1, *MYBPC3 + MYBPC3* = 1) and 10 were reclassified as a VUS (*MYBPC3 n* = 1, *MYH7 n* = 2, *RAF1 n* = 1, *TPM1 n* = 1, *ACTN n* = 2, *MYOM1 n* = 1, *PRKAG2 n* = 1, *PKP2 n* = 1). Three patients had more than one variant identified [*MYBPC3* (P) + *MYBPC3* (LP); *MYH7* (LP) + *PRKAG2* (LP); *MYH7* (LP) + *PKP2* (VUS)]. Baseline demographics for those with and without a disease‐causing sarcomeric variant identified on genetic testing is described in Supporting Information, *Table* *S5*.

For patients with a RASopathy following ACMG reclassification (*n* = 43), 33 patients had a single pathogenic/likely pathogenic variant (*RAF1 n* = 5, *PTPN11 n* = 17, *RIT1 n* = 6, *HRAS n* = 4, *BRAF n* = 1) and 5 had more than one variant [*PTPNP11* (P) + *MYH7* (VUS), *LZRT1* (VUS) + *MYH7* (VUS); *PTPN11* (P) + *MYH7* (LP); *LZRT1* (LP) + *HRAS* (VUS); *DSC2* (VUS) + *SCN5A* (VUS)].

### Clinical follow‐up

Median length of follow‐up was 4.1 years (IQR 1.3–8.1, range 0–30.9 years). Eighty‐nine patients were followed up for 10 years or longer [non‐syndromic HCM *n* = 43 (48.3%); RASopathy *n* = 41 (46.1%); IEM *n* = 5 (5.6%)].

### Interventions

*Table* *2* describes surgical and interventional procedures. An LV septal myectomy was performed in 28 patients (9.3%) (non‐syndromic *n* = 10, RASopathy *n* = 17, IEM *n* = 1) at median age 4.8 years (IQR 1.0–6.5, range 0–13.2), with concomitant mitral valve repair in 6. Twenty‐two patients (7.3%) (RASopathy *n* = 20, IEM *n* = 2) underwent RVOT obstruction relief (balloon pulmonary valvulopasty *n* = 9, surgical relief *n* = 18) at median age 7.3 months (IQR 4.1–10.8, range 0.8–112.3). Eight patients required more than one surgical procedure. Ten patients (3.3%) had an ICD inserted for primary (*n* = 6) or secondary (*n* = 3) prevention, at a median (range) age of 13.4 years (6.5–12.1) and 8.4 years (2.5–11.3), respectively, of which two had appropriate ICD therapies (Supporting Information, *Table* *S6*).

**Table 2:** Interventions (catheter and surgical) during follow‐up

Intervention			*N*
ICD	Primary		6
	Secondary		3
	Unknown indication		1
Pacemaker	Sinoatrial disease		2
	AV block		3
Surgery	RVOT relief		18
		RVOT Patch	11
		Valvotomy	4
		Suprapulmonary PS relief (patch)	1
		RVOT conduit	1
		Unknown	1
	Myectomy		28
		With MV repair	6
	Aortic valvotomy		3
	Other cardiac surgery	ASD closure	3
		PDA ligation	4
		Coarctation repair	2
		LVAD	2
		Sympathectomy	1
Multiple surgical procedures	Myectomy		2 (*n* = 3), 3 (*n* = 1)
	Mitral valve replacement		2 (previous MV plasty + myectomy; previous MV repair + RVOT relief)
	RVOT relief followed by myectomy		2
Catheter interventions	Balloon pulmonary valvuloplasty		9 (7 of whom required subsequent surgical intervention)
	PDA closure		1
EPS	Ablation		5 (symptomatic SVT *n* = 4, WPW *n* = 1)

ASD, atrial septal defect; AV, atrioventricular; EPS, electrophysiology study; ICD, implantable cardioverter defibrillator; LVAD, left ventricular assist device; MV, myectomy; PDA, patent ductus arteriosus; PS, pulmonary stenosis; RVOT, right ventricular outflow tract; SVT, supraventricular tachycardia; WPW, Wolff–Parkinson–White syndrome.

### Arrhythmias

Fifteen patients (4.9%) [non‐syndromic (*n* = 7, 5.1%); IEM (*n* = 5, 10.2%); RASopathy (*n* = 3, 3%)] had one or more MACE (sustained VT with haemodynamic compromise in eight; SCD in six; resuscitated cardiac arrest in four; and appropriate ICD therapy in two) during follow‐up (Supporting Information, *Table* *S6*), with an overall annual incidence rate of 0.88 [95% confidence interval (CI) 0.527–1.451]. Six MACE (43%) occurred during infancy; the remaining occurred at a mean age of 9.2 years (range 1.4–14.9). Eleven (3.7%) had supraventricular arrhythmias detected during follow‐up.

### Mortality

**Figure 2:**
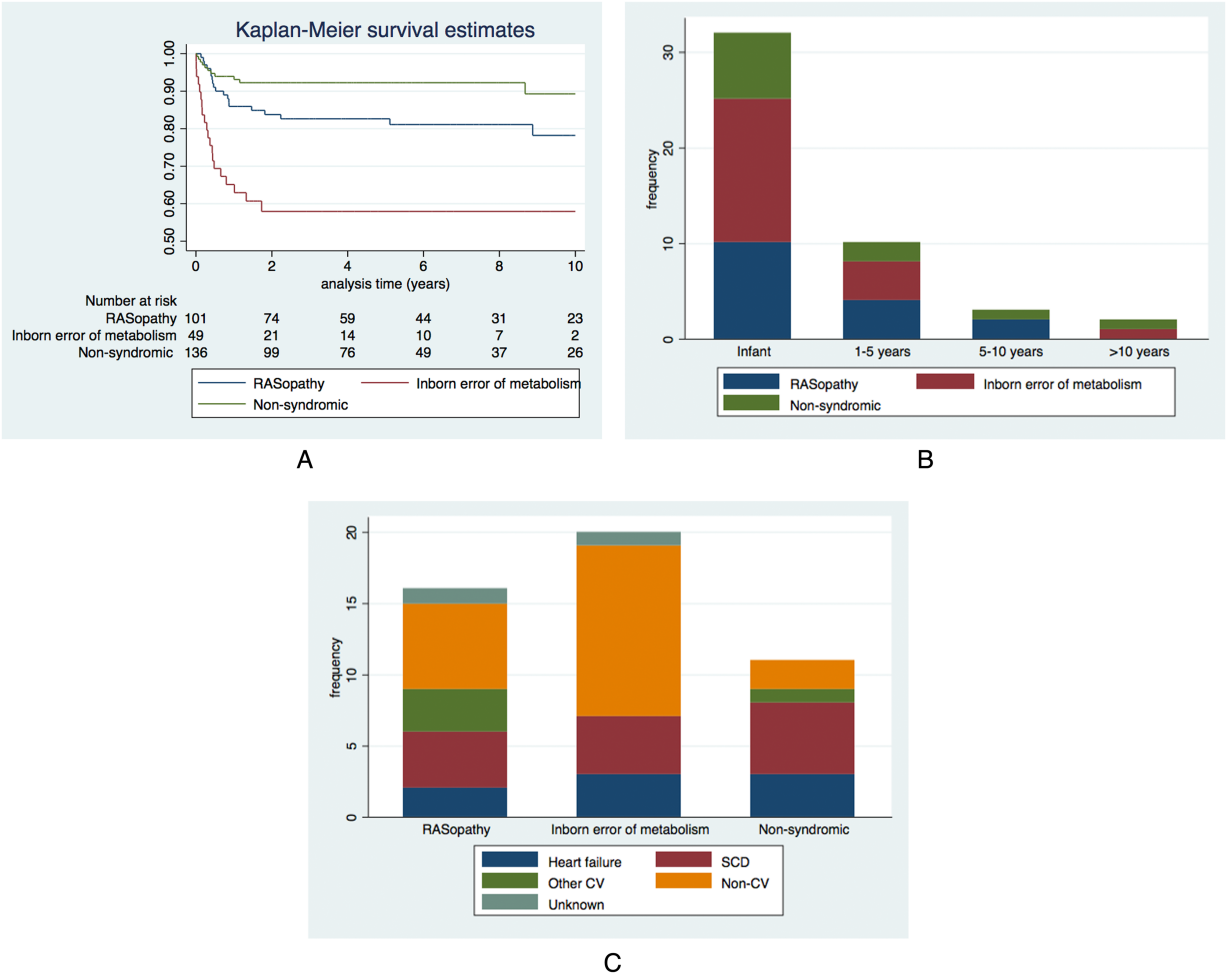
Long‐term survival of infantile hypertrophic cardiomyopathy. (A) Kaplan–Meier curves of transplant free survival by underlying aetiology. (B) Age at death by aetiology. (C) Cause of death by aetiology. CV, cardiovascular; SCD, sudden cardiac death.

A total of 253 patients (84.1%) were alive at last clinical follow‐up, including 6 (2.0%) who had undergone cardiac transplantation. Forty‐eight patients (15.9%) died: non‐CV 20 (6.6%) (infection *n* = 8, respiratory *n* = 3, metabolic acidosis *n* = 3, neurological insult *n* = 2, gastrointestinal bleed *n* = 1, not described *n* = 3); CCF 14 (4.7%); SCD 8 (2.7%); other CV 4 (1.3%); and unknown 2 (0.7%). Six patients (2%) were lost to clinical follow‐up. *Figure* *2* shows the cause and age at time of death by aetiology. Thirty‐two (66.7%) deaths occurred in infancy (IEM *n* = 15/23, 75.0%; RASopathy *n* = 10/16, 62.5%; non‐syndromic *n* = 7/11, 63.6%) most commonly secondary to non‐CV causes (IEM *n* = 9, 60%; RASopathy *n* = 4, 10%; non‐syndromic *n* = 1, 14.3%) or CCF (IEM *n* = 4, 26.7%; RASopathy *n* = 3, 30.0%; non‐syndromic *n* = 5, 71.4%). Of deaths occurring after early childhood (>5 years), four (80%) were sudden and one (20%) was heart failure related. Overall survival free from all‐cause mortality or transplant was 86.2% (81.7–89.7%) at 1 year and 83.1% (95% CI 78.2–87.0) at 5 years. Survival varied by aetiology (*Table* *1*, *Figure* *2* *C*). Predictors of all‐cause mortality at baseline on multivariable analysis were an underlying diagnosis of an IEM [hazard ratio (HR) 4.40 (1.95–9.66), *P* = 0.070], cardiac symptoms [HR 3.26 (95% CI 1.42–7.48), *P* = 0.005], and impaired LV systolic function [HR 2.97 (95% CI 1.12–7.87), *P* = 0.028]. Of those with impaired systolic function at baseline, 6 (50%) died; cause of death was SCD (*n* = 1), CCF (*n* = 1), and non‐CV death (sepsis *n* = 3, metabolic acidosis *n* = 1). Predictors of CV mortality or transplantation on multivariable analysis were cardiac symptoms at diagnosis [HR 19.1 (95% CI 4.22–86.70), *P* < 0.001] and higher MLVWT [HR 1.19 per mm increase (95% CI 1.07–1.34), *P* = 0.002] (*Table* *3*).

**Table 3:** Univariable and multivariable Cox regression analysis for predictors of outcome

		All‐cause mortality				Cardiovascular mortality or transplant			
		Univariable analysis		Multivariable analysis		Univariable analysis		Multivariable analysis	
		HR (95% CI)	*P* value	HR (95% CI)	*P* value	HR (95% CI)	*P* value	HR (95% CI)	*P* value
Female		0.94 (0.527–1.695)	0.849			0.85 (0.409–1.784)	0.675		
Aetiology	Non‐syndromic	Baseline				Baseline			
	RASopathy	1.87 (0.867–4.029)	0.110			1.10 (0.534–2.272)	0.793		
	IEM	6.40 (3.054–13.484)	<0.001	4.40 (1.950–9.659)	<0.001	2.45 (1.088–5.521)	0.029	2.19 (0.938–5.125)	0.070
Co‐morbidities	Cardiac	0.82 (0.406–1.645)	0.571			0.81 (0.328 0 1.983)	0.640		
	Any	0.63 (0.345–1.140)	0.126			1.29 (0.631–2.653)	0.482		
Family history of HCM		0.26 (0.095–0.735)	0.011			0.44 (0.155–1.280)	0.133		
Family history of SCD		1.11 (0.399–3.094)	0.840			1.32 (0.401–4.340)	0.649		
Any symptoms at baseline		3.96 (2.011–7.805)	<0.001	3.26 (1.419–7.483)	0.005	7.84 (2.727–22.557)	<0.001	19.1 (4.222–86.704)	<0.001
LVOTO		1.49 (0.775–2.852)	0.233			0.66 (0.314–1.385)	0.272		
RVOTO		1.37 (0.634–2.957)	0.424			2.07 (0.736–5.844)	0.167		
Increasing maximal wall thickness		1.01 (0.934–1.100)	0.742				0.0437	1.19 (1.067–1.344)	0.002
Impaired systolic function		5.43 (2.117–13.248)	<0.001	2.97 (1.124–7.865)	0.028	1.46 (0.190–11.217)	0.717		

CI, confidence interval; HCM, hypertrophic cardiomyopathy; HR, hazard ratio; IEM, inborn error of metabolism; LVOTO, left ventricular outflow tract obstruction; RVOTO, right ventricular outflow tract obstruction; SCD, sudden cardiac death.

## Discussion

This multicentre study of infantile HCM is, to our knowledge, the first systematic description of infant‐onset HCM and describes a complex and varied cohort of patients with a diverse phenotypic spectrum, clinical course, and outcome. Overall prognosis was poor, but was largely dependent on underlying aetiology, which emphasizes the importance of making an accurate diagnosis in these patients. Genetic testing identified a disease‐causing variant in up to 70% and should be considered for all patients with infant‐onset HCM.

### Aetiology of infantile hypertrophic cardiomyopathy

The aetiology of childhood HCM is more varied than that seen in adulthood, driven by a higher proportion of IEMs and RASopathies in patients presenting in infancy.^4^, ^5^, ^6^, ^9^ This is highlighted by the findings in this study, in which over 50% of patients had metabolic or syndromic disease. The proportion of patients with syndromic disease in particular was higher than previously reported in 328 patients with infantile HCM in the Pediatric Cardiomyopathy Registry (PCMR)^6^; this may reflect differences in systematic screening of children with syndromes known to be associated with HCM, or more comprehensive aetiological investigations in the expert centres recruiting to this study.

A major strength of this study is the high frequency of genetic testing and diagnostic yield, with an identifiable underlying aetiology identified in the majority of the patients, emphasizing the importance of systematic and comprehensive aetiological investigations, including genetics, in infantile HCM. Almost half the patients were classified as having non‐syndromic disease, and a disease‐causing sarcomeric variant was identified in 60% of those in whom genetic testing was performed most commonly in *MYBPC3* (*n* = 15) or *MYH7* (*n* = 14), supporting the notion that sarcomeric disease can manifest at any age.^23^, ^24^ The lack of significant differences between those with a sarcomeric variant and those non‐syndromic patients in whom genetic testing was not performed suggests that a substantial proportion of these are also likely to have variants in one or more cardiac sarcomere protein genes.^25^, ^26^ Variants in the RAS‐MAPK pathway genes were identified in three non‐syndromic patients, in keeping with previous reports that non‐sarcomeric variants may contribute to the disease phenotype in children with apparently non‐syndromic disease.^8^ Additionally, one patient with metabolic disease had a co‐existing pathogenic sarcomeric variant, which emphasizes the importance of considering dual pathology. Although case reports and small case series have described severe early‐onset disease associated with compound or heterozygote sarcomeric gene variants,^27^, ^28^ only one patient had a compound heterozygote variant in MYBPC3 and two further patients had an additional VUS in a cardiomyopathy‐associated gene. It is possible that compound heterozygosity or double homozygosity results in foetal demise in most cases, but these findings suggest that the majority of disease seen in infancy is caused by single pathogenic sarcomeric variants. The presence of variants in non‐HCM genes, although unlikely to explain the HCM phenotype alone, could act as modifiers for other, as yet unidentified, HCM disease‐causing variants. Previous studies have suggested that patients with *de novo* sarcomeric variants are at higher risk of adverse outcomes, including mortality. Data on inheritance pattern were not collected in this study, but future studies systematically investigating the prevalence of *de novo* variants and role of genotype in long‐term outcomes for infantile HCM would be useful.

A family history of HCM was present in a higher proportion of non‐syndromic patients than expected (38%), one quarter of patients with an IEM, and nearly 1 in 10 patients with a RASopathy syndrome, highlighting the need for performing family screening regardless of the aetiology.^23^, ^24^

### Phenotypic characteristics

A major finding of this study is the high frequency of associated complex medical needs, with over half having an additional co‐morbidity. The most frequent co‐morbidities were cardiac or neurological, but this differed by underlying aetiology. As expected, co‐morbidities were most common in those with syndromic disease, but were also present in one‐third of those classified with non‐syndromic disease. It is possible that a proportion of the so‐called ‘non‐syndromic’ patients had undiagnosed syndromic disease as genetic testing was not universally performed. However, there was no difference in the prevalence of co‐morbidities between those undergoing genetic testing or those identified to have a disease‐causing sarcomere variant suggesting that co‐morbidities are also common in non‐syndromic infantile HCM patients. It is noteworthy that one‐third of patients with an IEM had no additional co‐morbidities; this could reflect under‐reporting of non‐cardiac features by cardiologists, a predominant cardiac phenotype in some IEM, or age‐related penetrance of non‐cardiac manifestations.

Consistent with previous reports, the HCM phenotype varied according to the underlying aetiology.^5^, ^6^, ^10^, ^13^ Concentric LV hypertrophy and biventricular hypertrophy were more common in those with syndromic disease; co‐existing RVOT obstruction was seen predominantly in RASopathy patients, and LVOT obstruction was rare in those with IEM. One‐third of patients had heart failure symptoms at presentation, and 12 (8%) had impaired LV systolic function, the majority of which (*n* = 7) had an IEM. Outlook was poor for this subgroup of patients, with 50% dying during follow‐up, although the majority from non‐cardiac causes. The finding of impaired systolic function should therefore prompt clinicians to look for an underlying syndromic or metabolic aetiology.

### Long‐term outcomes

Diagnosis of HCM in infancy has been shown repeatedly to be associated with poor short‐term outcomes,^10^ and in keeping with this, in our cohort, 1 year mortality was 14%. However, survival rates varied significantly according to the underlying aetiology, with non‐syndromic patients having a much better prognosis compared with IEM. The cause of death also differed by aetiology, with non‐CV causes accounting for the majority of deaths for IEM, whilst cardiac causes were more common for non‐syndromic HCM or RASopathies. One strength of this study is the long‐term follow‐up of patients (30% had over 10 years' follow‐up), allowing long‐term trends in survival to be investigated. For all aetiology groups, most deaths occurred during infancy, with survival curves plateauing in later follow‐up, suggesting that, for those infants who survive beyond 2 years, the prognosis is substantially better. Previous long‐term population studies have described differences in the mode of death during follow‐up,^9^ with early deaths caused by heart failure and later deaths resulting from ventricular arrhythmias. Whilst similar trends were seen in this study, the majority of early deaths were non‐cardiac and 3 out of 11 arrhythmic deaths occurred during infancy. This highlights both the importance of risk stratification for SCD at all ages and the burden of non‐CV disease in infantile HCM.

The overall MACE rate (0.82/100 patient years) seen in this cohort was lower than that reported in children presenting above 1 year (approximately 1.2–1.4/100 patient years).^4^, ^6^, ^9^, ^29^ Importantly, over half of MACE were in patients with syndromic disease, including IEM (e.g. Pompe's and mitochondrial) and RASopathies, the majority of which occurred in infancy, challenging the concept that malignant arrhythmias are rare in syndromic and metabolic HCM. It is beyond the scope of this study to investigate risk stratification for SCD in infant‐onset disease, and further work to identify risk factors specific to the infantile HCM population is required.

Heart failure symptoms,^10^, ^11^, ^13^ low birth weight,^10^ degree of hypertrophy,^5^, ^9^, ^10^, ^11^ impaired systolic function,^9^, ^11^ ‘mixed’ phenotypes,^10^ and underlying aetiology^9^, ^11^ have been described as important clinical predictors of worse outcomes. However, most studies to date have been limited by small patient numbers and a lack of detailed aetiological information, with infantile disease treated as a homogenous group for the purpose of analysis. In our cohort, all‐cause mortality and CV mortality or transplantation were associated not only with the presence of symptoms and echocardiographic phenotypic parameters but also with a diagnosis of IEM. This suggests that underlying aetiology as well as phenotype is important for prognosis and emphasizes the importance of a systematic approach to making an accurate diagnosis in this group of patients.

### Limitations

This study is limited by inherent problems of retrospective studies, in particular missing or incomplete data, particularly in relation to genetic variant data. Variations in clinical assessment and patient management are inevitable as patients were recruited from multiple centres in different geographical locations. As genetic testing was performed at the participating clinicians' discretion and across different eras with different sized panels and gene sequencing techniques, it is beyond the scope of this study to discuss the clinical yield of genetic testing in infantile HCM. Although a high proportion of patients with a RASopathy syndrome or IEM had a disease‐causing variant identified on genetic testing, it is not known whether genetic testing results altered the final diagnosis or confirmed previous clinical suspicions. Further work to explore the age‐related and gene‐related penetrance of sarcomeric disease in infant HCM is needed. Although the mortality rate is unlikely to be affected by these missing data due to nationally recorded death data in participating countries, other outcomes, such as arrhythmic events or surgical interventions, could have been underestimated.

## Conclusions

This large multicentre study of infantile HCM describes a complex and varied cohort of patients with a diverse phenotypic spectrum, genetic substrate, clinical course, and outcome. Prognosis depends on clinical presentation, disease phenotype, and the underlying aetiology, which emphasizes the importance of making an accurate diagnosis in these patients. Genetic testing identified a disease‐causing variant in up to 70% and should be considered for all patients with infant‐onset HCM. Arrhythmic events were rare but occurred during infancy and in patients with syndromic disease, highlighting the importance of a systematic approach to diagnosis, screening, and risk stratification even in very young patients with HCM.

## Conflict of interest

None to declare.

## Funding

This work was supported by the British Heart Foundation (grant number FS/16/72/32270) to G.N. and J.P.K. and the Association for European Paediatric and Congenital Cardiology (AEPC junior grant) to G.N. J.P.K. and E.F. are supported by Max's Foundation, Great Ormond Street Hospital Charity, and Great Ormond Street Hospital for Children. J.P.K. is the recipient of a Medical Research Council (MRC) Clinical Academic Research Partnership (CARP) award. This work is (partly) funded by the NIHR GOSH BRC. The views expressed are those of the author(s) and not necessarily those of the NHS, NIHR, or the Department of Health. This work was (partly) supported by Children's Memorial Health Institute (statutory grant number S176/2018) to L.Z.

## Supporting information


**Table S1.** Aetiology of patients with infantile disease.
**Table S2.** Prevalence of comorbidities by underlying diagnosis.
**Table S3.** Use of genetic testing over time by underlying aaetiology.
**Table S4.** Results of genetic testing in non‐syndromic and RASopathy patients.
**Table S5.** Comparison of baseline demographics in Non‐syndromic patients according to genetic testing status.
**Table S6.** Description of clinical characteristics and outcomes of patients with major arrhythmic cardiac events.
**Figure S1.** Comorbidities according to aetiology in infantile HCM
**Figure S2.** Genetic testing strategy in infantile HCM
