# Supplementary material for: Clinical presentation and long‐term outcomes of infantile hypertrophic cardiomyopathy: a European multicentre study
Source: ESC Heart Fail. 2021 Sep 6;8(6):5057–67. doi: 10.1002/ehf2.13573 (PMC8712843; doi:10.1002/ehf2.13573)
Supplement: Supplementary file 1 — Table S1. Aetiology of patients with infantile disease. Table S2. Prevalence of comorbidities by underlying diagnosis. Table S3. Use of genetic testing over time by underlying aaetiology. Table S4. Results of genetic testing in non‐syndromic and RASopathy patients. Table S5. Comparison of baseline demographics in Non‐syndromic patients according to genetic testing status. Table S6. Description of clinical characteristics and outcomes of patients with major arrhythmic cardiac events. Figure S1. Comorbidities according to aetiology in infantile HCM Figure S2. Genetic testing strategy in infantile HCM [file EHF2-8-5057-s001.docx]

**Supplementary files**

**Supplementary table 1: Aetiology of patients with infantile disease**

| **Aetiology** | **RASopathy** | | | **101 (33.6%)** |
| --- | --- | --- | --- | --- |
|  |  | Noonan syndrome | | 81 (26.9%) |
|  |  | Costello syndrome | | 10 (3.3%) |
|  |  | NSML | | 9 (2.9%) |
|  |  | Unknown | | 1 (0.3%) |
|  | **Inborn error of metabolism** | | | **49 (16.3%)** |
|  |  | Mitochondrial |  | 14 |
|  |  |  | TMEM70 deficiency | 2 |
|  |  |  | Complex 1 deficiency | 5 |
|  |  |  | Complex IV deficiency | 1 |
|  |  |  | Combined oxidative phosphorylation deficiency 10 (MTO1) | 1 |
|  |  |  | Mitochondrial encephalopathy | 1 |
|  |  |  | Leigh disease | 1 |
|  |  |  | Sengers syndrome | 1 |
|  |  |  | Unknown | 2 |
|  |  | Disorders of carbohydrate metabolism |  | 20 |
|  |  |  | Carbohydrate glycoprotein deficiency | 2 |
|  |  |  | Pompe | 16 |
|  |  |  | Glycogen storage Disorder type III | 2 |
|  |  | Urea cycle defects | OTC deficiency | 1 |
|  |  | Disorders of amino acid metabolism | Sulphite oxidase deficiency | 1 |
|  |  | Lysosomal storage diseases | Danon disease | 1 |
|  |  | Disorders of fatty acid oxidation | VLCAD | 2 |
|  |  | Other | congenital generalised lipodystrophy | 3 |
|  |  |  | 3 methyl glutonic aciduria | 1 |
|  |  |  | no unifying diagnosis | 6 |
|  | **Neuromuscular syndrome** | | | **1 (0.3%)** |
|  |  | Emery Dreifus MD | | 1 (0.3%) |
|  | **Non-syndromic** | | | **138 (45.6%)** |
|  | **Other** | | | **12 (4%)** |
|  |  | Timothy Syndrome |  | 1 |
|  |  | William syndrome |  | 1 |
|  |  | Kabuki |  | 1 |
|  |  | Aicardi Goutier |  | 1 |
|  |  | NF1 |  | 1 |
|  |  | CHARGE syndrome |  | 1 |
|  |  | Multi-system involvement with no unifying diagnosis |  | 6 |

**Supplementary table 2: Prevalence of comorbidities by underlying diagnosis**

|  | Non-syndromic (n=138) | RASopathy (n=101) | IEM (n=49) | P value |  |  |  |  |
| --- | --- | --- | --- | --- | --- | --- | --- | --- |
| Any comorbidity | 50 (36.2%) | 76 (75.3%) | 35 (71.4%) | <0.001 |  |  |  |  |
| Other cardiac defects+ | 30 (21.7%) | 42 (41.6%) | 4 (8.2%) | <0.001 |  |  |  |  |
| Neurological/neuromuscular* | 18 (13.0%) | 29 (28.7%) | 25 (51.0%) | <0.001 |  |  |  |  |
| Genitourinary | 0 | 15 (14.9%) | 2 (4.1%) | <0.001 |  |  |  |  |
| Respiratory/ENT | 3 (2.2%) | 8 (16.3%) | 21 (42.9%) | <0.001 |  |  |  |  |
| Gastrointestinal | 5 (3.6%) | 9 (8.9%) | 8 (16.3%) | 0.012 |  |  |  |  |
| Orthopaedic | 3 (2.2%) | 6 (5.9%) | 3 (6.1%) | 0.216 |  |  |  |  |
| Malignancy | 0 | 1 (1%) | 0 | 0.521 |  |  |  |  |
| Endocrine | 1 (0.7%) | 4 (4%) | 1 (2%) | 0.223 |  |  |  |  |
| Haematological | 1 (0.7%) | 2 (4.1%) | 4 (4.0%) | 0.128 |  |  |  |  |
| Other comorbidity | 0 | 5 (5.0%) | 3 (6.1%) | 0.006 |  |  |  |  |

*includes autistic spectrum disorders, ADHD

+ cardiac comorbidities (pulmonary stenosis n=22, septal defects n=35, patent ductus arteriosus n=6, left ventricular outflow tract obstruction n=9 [coarctation, shone complex, bicuspid aortic valve), Long QT syndrome n=3, Wolff-Parkinson White syndrome, n=2, SVT n=1).

**Supplementary table 3: Use of genetic testing over time by underlying aaetiology**

|  | | Whole cohort (n=301) | RASopathy (n=101) | IEM (n=49) | Non-syndromic (n=138) |
| --- | --- | --- | --- | --- | --- |
| Genetic testing performed | | 163 (54.2%) | 72 (71.3%) | 24 (49.0 %) | 67 (48.6%) |
|  | Pre-2000 | 11 (57.9%) | 6 (66.8%) | 0 | 5 (55.6%) |
|  | 2000-2005 | 9 (40.9%) | 2 (22.2%) | 0 | 7 (58.3%) |
|  | 2005-2010 | 29 (49.2%) | 14 (70%) | 5 (38.5%) | 10 (38.5%) |
|  | 2010-2015 | 70 (63.1%) | 32 (78.1%) | 9 (47.4%) | 29 (56.9%) |
|  | 2015 + | 44 (57.1%) | 18 (81.2%) | 10 (66.8%) | 16 (40.0%) |
|  | P value for era change | 0.244 | 0.0012 | 0.355 | 0.370 |

**Supplementary table 4: Results of genetic testing in non-syndromic and RASopathy patients**

| Non-syndromic (n=138) | | | | |
| --- | --- | --- | --- | --- |
| Genetics performed | | | | 67 (48.6%) |
|  | No variant identified | | | 22 (32.8%) |
|  | Variant identified | | | 40 (60%) |
|  | Pending | | | 5 (7.5%) |
|  | | **Single variant** | | **37** |
|  |  |  | *MYBPC3* | 15 |
|  |  |  | *MYH7* | 14 |
|  |  |  | *TPM1* | 1 |
|  |  |  | *ACTC1* | 1 |
|  |  |  | *ACTN2* | 1 |
|  |  |  | *KRAS* | 1 |
|  |  |  | *LZTR1* | 1 |
|  |  |  | *RAF1* | 1 |
|  |  |  | *SCN5a* | 1 |
|  |  |  | *MYOM1* | 1 |
|  |  | **Two variants** | | **3** |
|  |  |  | *MYBPC3 + MYBPC3* | 1 |
|  |  |  | *MYH7 + PRKAG2* | 1 |
|  |  |  | *MYH7 + PKP2* | 1 |
| RASopathy (n=101) | | | | |
| Genetics performed | | | | 72 (71.3%) |
|  | No variant identified | | | 5 (6.9%) |
|  | Variant identified | | | 59 (81.9%) |
|  | Result unknown | | | 2 (2.8%) |
|  | Pending | | | 6 (8.3%) |
|  | | **Single variant** | | **54** |
|  |  |  | *RAF1* | 12 |
|  |  |  | *PTPN11* | 22 |
|  |  |  | *RIT1* | 7 |
|  |  |  | *HRAS* | 5 |
|  |  |  | *BRAF* | 1 |
|  |  |  | *KRAS* | 2 |
|  |  |  | *SHOC2* | 1 |
|  |  |  | *SOS* | 4 |
|  |  | **Two variants** | | **5** |
|  |  |  | *PTPN11 + MYH7* | 1 |
|  |  |  | *LZRT1 + MYH7* | 1 |
|  |  |  | *PTPN11 + MYH7* | 1 |
|  |  |  | *LZRT1 + HRAS* | 1 |
|  |  |  | *DSC2 + SCN5a* | 1 |
| IEM (n=49) | |  |  |  |
| Genetics performed | | | | 23 (46.9%) |
|  | No variant identified | | | 4 (17.4%) |
|  | Variant identified | | | 16 (69.6%) |
|  | Pending | | | 3 (13%) |
|  | | | *BSCL2* | 3 |
|  |  |  | *GAA* | 3 |
|  |  |  | *ACAD9* | 2 |
|  |  |  | *TMEM70* | 3 |
|  |  |  | *AGK* | 1 |
|  |  |  | *AGL* | 1 |
|  |  |  | *LAMP2* | 1 |
|  |  |  | *MTO1* | 1 |
|  |  |  | *MYH7* | 1 |

**Supplementary table 5: Comparison of baseline demographics in Non-syndromic patients according to genetic testing status**

|  |  | Genetic testing (n=67) | No genetic testing (n=71) | | P value | | Sarcomere gene positive (n=40) | | Sarcomere gene negative (n=22) | P value | | |
| --- | --- | --- | --- | --- | --- | --- | --- | --- | --- | --- | --- | --- |
| Male gender | | 43 (64.2%) | 45 (63.4%) | | 0.922 | | 25 (62.5%) | | 14 (63.6%) | 0.929 | | |
| FHx HCM (n=128) | | 33 (49.3%) | 15 (24.6%) | | **0.004** | | 25 (62.5%) | | 7 (31.8%) | **0.033** | | |
| FHx SCD (n=122) | | 7 (10.9%) | 6 (10.3%) | | >0.999 | | 4 (10.8%) | | 2 (9.1%) | 0.833 | | |
| Co-morbidities | Any | 22 (32.8%) | 28 (39.4%) | | 0.420 | | 12 (30.0%) | | 7 (31.8%) | 0.882 | | |
|  | Cardiac | 13 (19.4%) | 17 (23.9%) | | 0.543 | | 8 (20.0%) | | 2 (9.1%) | 0.472 | | |
| NYHA >2 (n=112) | | 11 (17.2%) | 113 (27.1) | | 0.207 | | 4 (10.8%) | | 7 (31.8%) | 0.081 | | |
| Any cardiac medication at baseline (n=120) | | 33 (54.1%) | 27 (45.8%) | | 0.361 | | 16 (45.7%) | | 15 (68.2%) | 0.111 | | |
| Pattern of hypertrophy (n=262) | ASH | 53 (82.8%) | 27 (46.6%) | | **<0.001** | | 32 (84.2%) | | 17 (81.0%) | 0.866 | | |
|  | Concentric | 4 (6.3%) | 12 (20.7%) | |  |  | 2 (5.3%) | | 2 (9.5%) |  |  |  |
|  | Biventricular | 7 (10.9%) | 15 (25.9%) | |  |  | 4 (10.5%) | | 2 (9.5%) |  |  |  |
| MWT (mm) | Median, IQR | 10.5 (8, 13) | 8.5 (4.9, 12.0) | | 0.108 | | 10 (8, 12) | | 12 (9, 15) | 0.1088 | | |
| MWT z score | Mean (+/-) | 11.6 (+/-5.3) | 9.9 (+/-6.1) | | 0.126 | | 10.5 (+/-4.5) | | 14.1 (+/-6.0) | **0.029** | | |
| Impaired systolic function (n=138) | | 1 (1.5%) | 2 (2.8%) | | >0.999 | | 0 | | 1 (4.6%) | 0.355 | | |
| LVOT obstruction (n=117) | | 22 (36.1%) | 20 (35.7%) | | 0.968 | | 9 (25.0%) | | 11 (52.4%) | **0.048** | | |
| RVOT obstruction (>16mmHg) | | 6 (26.1%) | 6 (46.2%) | | 0.220 | | 2 (13.3%) | | 5 (50.0%) | 0.131 | | |
| Died | | 3 (4.5%) | 8 (11.3%) | | 0.210 | | 0 | | 3 (13.6%) | **0.041** | | |
| SCD | | 2 (3%) | 1 (1.4%) | |  |  | 0 | | 2 (9.1%) |  |  |  |
| CCF | | 1 (1.5%) | 5 (7.0%) | |  |  | 0 | | 1 |  |  |  |
| Non-CV | | 0 | 2 (2.8%) | |  |  | 0 | | 0 |  |  |  |
| Transplant | | 1 (1.4%) | 1 (1.5%) | | NA | | 1 | | 0 | NA | | |
| Mortality or transplant incidence rate/100 patient years | | | 0.87  (95% CI 0.33 – 2.32) | 2.44  (95% CI 1.27 – 4.69) | 0.1005 | |  | |  | | |  |
| MACE | | 6 (9.0%) | 1 (1.4%) | | **0.046** | | 3 (7.5%) | | 3 (13.6%) | 0.657 | | |
| MACE incidence rate/100 patient years | | 1.13  (95% CI 0.47 – 2.70) | 0.27  (95% CI 0.04-1.92) | | 0.1716 | |  | |  |  | | |

Sarcomere gene positive defined as centre reported variant of unknown significance or disease causing variant in a sarcomeric protein gene.

**Supplementary table 6: Description of clinical characteristics and outcomes of patients with major arrhythmic cardiac events**

|  | Diagnosis | Age at event | Event | Presentation | Patient outcome |
| --- | --- | --- | --- | --- | --- |
| 1 – 168 | RASopathy – NSML syndrome | 2 months | Sustained VT | Referred for heart failure symptoms, MWT 18mm, BVH, biventricular outflow tract obstruction | Referred for Cardiac transplant  Died CCF aged 0.29 |
| 2 – 233 | Non-syndromic | 4 months | VF arrest | Presented with VF arrest. MWT 6mm, concentric, impaired systolic function | Recurrent ventricular arrhythmias.  Died intractable VT/VF aged 4 months |
| 3 – 137 | IEM – Mitochondrial (Complex 1 deficiency) | 4 months | Sustained VT | Presented with multi-organ failure. MWT 13mm, BVH. No OTO | Died intractable arrhythmias aged 0.44years |
| 4 – 260 | IEM – no unifying diagnosis | 3 months | Aborted cardiac arrest | Referred for heart failure symptoms, MWT 16, BVH | Pacemaker inserted for AV block aged 3 months  Died aged 6 months SCD |
| 5- 136 | IEM - Pompe | 5months | VF arrest during GA intubation | Referred for heart failure symptoms, MWT 19mm, BVH, Impaired systolic fx | Died (sepsis, PEA arrest) aged 6 months |
| 6 – 256 | IEM - Pompe | 5 month | Aborted cardiac arrest | Referred for heart failure symptoms, MWT 10mm, BVH | Died CCF 6 months |
| 7 – 141 | RASopathy - Noonan | 16 months | Sudden cardiac death | Referred for heart failure symptoms, MWT 12mm, BVH, RVOTO | Listed for cardiac tx. Sustained VT died aged 16 months |
| 8 – 269 | Non-syndromic | 2 years, 5 mo | Aborted cardiac arrest | Referred for murmur. MWT 8mm, ASH | Secondary prevention ICD inserted. Subsequent appropriate therapies  Underwent sympathectomy aged 3  Alive and well at last follow up (3.2 years) |
| 9 – 166 | Non-syndromic | 8 years | Sudden cardiac death | MWT 12mm, BVH, LVOTO | Myectomy + MV repair aged 5. Residual LVOTO post-op  SCD aged 8 |
| 10 – 274 | RASopathy - Noonan | 8 years | Sudden cardiac death | Referred for heart failure symptoms, MWT 10mm. | Myectomy aged 6 |
| 11 - 265 | Non-syndromic | 12 years | Sudden cardiac death | Referred for heart failure symptoms, MWT8mm ASH, LVOTO | Myectomy aged 2 |
| 12 – 25 | IEM – no unifying diagnosis (likely storage disorder) | 14 years | Sudden cardiac death | Pre-excitation on ECG, MWT 9mm concentric, | Syncope aged 13 years, Reveal inserted  Died (VT -> VF) aged 14 |
| 13 – 250 | Non-syndromic | 12 years | Aborted cardiac arrest | FHx HCM, presented with SOB | VF arrest on exercise. Secondary prevention ICD. Multiple appropriate therapies for VT. Underwent cardiac tx aged 15 |
| 14 – 213 | Non-syndromic | 16 years | Sustained VT | No symptoms at presentation. FHx HCM, MWT 11mm ASH | ICD implantation for primary prevention aged 7 years  Alive and well at last follow up (17 years) |
| 15 - 100 | Non-syndromic | 7 years | Sustained VF post-myectomy | Family screening. No symptoms at presentation. MWT 10mm, concentric. LVOTO | Myectomy aged 7. Primary prevention ICD aged 8.  Alive and well aged 17 |

NSML = Noonan syndrome with multiple lentigines, VT = ventricular tachycardia, MWT = maximal wall thickness, BVH = biventricular ventricular hypertrophy, CCF = congestive cardiac failure, VF = ventricular fibrillation, IEM = inborn error of metabolism, OTO = outflow tract obstruction, AV = atrioventricular GA = general anaesthesia, PEA = pulseless electrical activity, RVOTO = right ventricular outflow tract obstruction, ASH = asymmetric septal hypertrophy, ICD = implantable cardioverter defibrillator, MV = mitral valve, LVOTO = left ventricular outflow tract obstruction, FHx = family history of, SOB = shortness of breath

Supplementary Figure 1: Comorbidities according to aetiology in infantile HCM


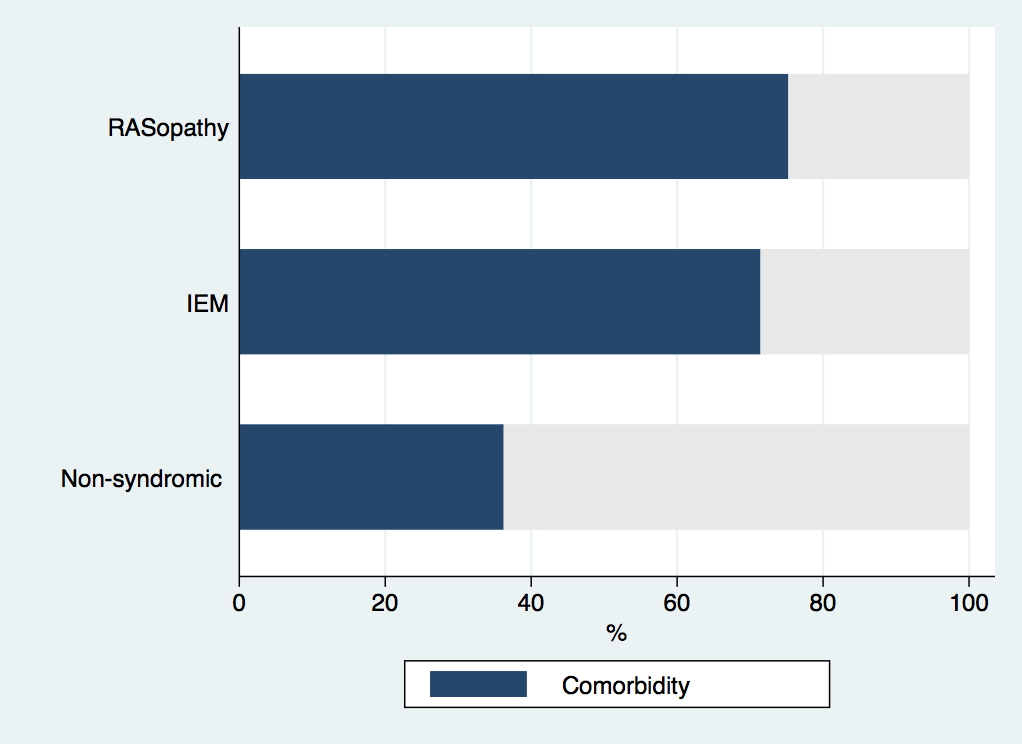


Supplementary Figure 2: Genetic testing strategy in infantile HCM
